# Identification of the Active Ingredient and Beneficial Effects of *Vitex rotundifolia* Fruits on Menopausal Symptoms in Ovariectomized Rats

**DOI:** 10.3390/biom11071033

**Published:** 2021-07-16

**Authors:** Ji Hwan Lee, Sullim Lee, Quynh Nhu Nguyen, Hung Manh Phung, Myoung-Sook Shin, Jae-Yong Kim, Hyukjae Choi, Sang Hee Shim, Ki Sung Kang

**Affiliations:** 1 College of Korean Medicine, Gachon University, Seongnam 13120, Korea; kleert26@gmail.com (J.H.L.); quynhnhunguyen.nnq@gmail.com (Q.N.N.); manhspkt92@gmail.com (H.M.P.); ms.shin@gachon.ac.kr (M.-S.S.); 2 Department of Life Science, College of Bio-Nano Technology, Gachon University, Seongnam 13120, Korea; sullimlee@gachon.ac.kr; 3 College of Pharmacy, Duksung Women’s University, Seoul 01369, Korea; kjaey0331@naver.com; 4 College of Pharmacy, Yeungnam University, Gyeongsan 38541, Korea; h5choi@yu.ac.kr; 5 Natural Products Research Institute, College of Pharmacy, Seoul National University, Gwanak-ro, Gwanak-gu, Seoul 08826, Korea

**Keywords:** *Vitex rotundifolia*, menopause, ovariectomized rat, E-screen, MCF-7 cell

## Abstract

Estrogen replacement therapy is a treatment to relieve the symptoms of menopause. Many studies suggest that natural bioactive ingredients from plants resemble estrogen in structure and biological functions and can relieve symptoms of menopause. The fruit of *V. rotundifolia*, called “Man HyungJa” in Korean, is a traditional medicine used to treat headache, migraine, eye pain, neuralgia, and premenstrual syndrome in Korea and China. The aim of the present study was to confirm that *V. rotundifolia* fruit extract (VFE) exerts biological functions similar to those of estrogen in menopausal syndrome. We investigated its in vitro effects on MCF-7 cells and in vivo estrogen-like effects on weight gain and uterine contraction in ovariectomized rats. Using the polar extract, the active constituents of VFE (artemetin, vitexicarpin, hesperidin, luteolin, vitexin, and vanillic acid) with estrogen-like activity were identified in MCF-7 cells. In animal experiments, the efficacy of VFE in ameliorating body weight gain was similar to that of estrogen, as evidenced from improvements in uterine atrophy. Vitexin and vitexicarpin are suggested as the active constituents of *V. rotundifolia* fruits.

## 1. Introduction

Menopause is a permanent cessation state of the natural female reproductive life resulting from the loss of ovarian follicular activity [1]. In most women, menopause begins in mid-to-late 40 years of age and gradually progresses. Postmenopausal women suffer from many menopausal symptoms such as depression, mood swings, hot flashes, and vaginal dryness, which are caused by hormonal imbalances, especially by the decrease in estrogen level [2]. Postmenopausal women are more interested in menopausal symptoms, quality of life, and health after menopause.

Estrogen, which is a hormone mainly produced in the ovaries, plays multiple biological functions to control energy homeostasis, core body temperature, and bone remodeling. Estrogen is known to play an important function in the development and maintenance of the reproductive system. Estrogen acts via nuclear steroid receptors such as estrogen receptor alpha (ER-α) and estrogen receptor beta (ER-β), which mediates the modulation of gene expression [3,4]. Estrogen binds to specific receptors and creates a ligand-receptor complex and fast non-genomic signaling of steroid hormones and ligand-independent action [5,6]. Non-genomic action of sex steroids has been shown in many studies on immediately responses following administration of 17β- estradiol (E2) to an ovariectomized mice model [7,8,9]. After menopause, estrogen is produced in small amounts through aromatase from adrenal androgen [10]. Menopausal hormone therapy provides relief from loss of bone density and bone fractures as well as the genitourinary syndrome of menopause [11]. Many recent studies have shown that hormone replacement therapy is associated with several benefits (lower risk for CHD, cancer, fractures, and overall mortality) and only rare risks (mainly deep vein thrombosis) for 50–60 years old women, but with fewer benefits and many increased risks for women aged more than 60 years [12,13,14,15]. Hormone replacement therapy relieves the symptoms of menopause. Many studies suggest that natural bioactive compounds from plants resemble estrogen in structure and biological functions and can relieve the symptoms of menopause [16]. Estrogenic compounds have been identified in flax and sesame seeds [17], wild yam [18], and soybean [19]. Genistein extracted from soybean has been extensively studied and known to relieve menopausal symptoms [20,21].

*Vitex rotundifolia* (Verbenaceae family) is a branched shrub that grows on beaches, rocky shorelines, and sand dunes and is widely distributed, particularly in the seashores of Korea. The fruit of *V. rotundifolia*, called “*Man HyungJa*” in Korean, is known as a traditional medicine used for the treatment of headache, migraine, eye pain, neuralgia, and premenstrual syndrome in Korea and China [22]. To date, diverse classes of compounds, including iridoids, phenylpropanoids, flavonoids, lignans, and diterpenes, have been reported from the fruit of *V. rotundifolia* [23]. Among these compounds, vitexicarpin is known to exhibit different pharmacological properties, including anti-proliferative, anti-inflammatory, neuroprotective, and analgesic activities [24]. Many studies have been conducted to show that flavonoids act similar to female hormones and are beneficial to menopausal women [17,18,19,20,21]. *V. rotundifolia* is known to contain many flavonoids, which may be beneficial to menopausal women. Although *V. rotundifolia* extracts and their constituents have various pharmacological effects, their estrogenic activities have not yet been elucidated. Therefore, we have evaluated the estrogen-like activity of the ethanol extract of the fruits of *V. rotundifolia* and its components, artemetin, vitexicarpin, hesperidin, luteolin, vitexin, and vanillic acid in this study.

## 2. Materials and Methods

### 2.1. Cells and Cell Culture

The MCF-7 cell line, which is an ER-positive human breast cancer cell line, was purchased from the American Type Culture Collection (ATCC, Manassas, VA, USA). MCF-7 cells were maintained in Roswell Park Memorial Institute (RPMI)-1640 medium (Corning, Manassas, VA, USA) supplemented with 10% fetal bovine serum (FBS; Gibco BRL, Carlsbad, MD, USA) at 37 °C in a humid atmosphere containing 5% CO_2_. In cell experiments, dimethyl sulfoxide (DMSO; Biosesang, Gyeonggi, Korea) was used as a vehicle to dissolve samples. The final DMSO concentration was under 0.1%, which is considered non-cytotoxic.

### 2.2. Plant Materials

The dried fruits of *V. rotundifolia* were purchased from Kyungdong Oriental Market (Seoul, South Korea) in October 2018 and botanically identified by one of the authors (S. H. Shim). A voucher specimen was deposited at the Natural Products Research Institute of the College of Pharmacy, Seoul National University (specimen No. NPC 21-1). The dried samples (30 g) were extracted thrice with 1 L of 30% aqueous ethanol (EtOH) for 1 h at 50 °C and the solvents were evaporated in vacuo at 40 °C to yield the extract (1.5 g).

### 2.3. Standards and Reagents

The standard compounds artemetin (1), vitexicarpin (2), hesperidin (3), luteolin (4), vitexin (5), and vanillic acid (6) used in this study were previously isolated from *V. rotundifolia* fruit extract (VFE) by emeritus Professor Sam Sik Kang from Seoul National University. Before the chemical and biological experiments conducted for this study, all the compounds were evaluated for their purities using thin-layer chromatography (TLC) and high-performance liquid chromatography (HPLC). The identity of all compounds was re-confirmed by nuclear magnetic resonance (NMR) and mass spectrometry (MS).

### 2.4. E-Screen Assay 

The estrogen-like activities of samples on MCF-7 cells were evaluated by a modified method of Soto’s E-screen assay [25]. In brief, 1 × 10^4^ MCF-7 cells were seeded into each well of a 48-well plate. After incubation for 24 h, the cells were treated with indicated concentrations of samples or DMSO (vehicle) in phenol red-free RPMI-1640 medium (Gibco, Carlsbad, CA, USA) containing 10% charcoal dextran stripped serum (Innovative Research, Novi, USA). At the same time, the antagonistic test groups were treated with the ER antagonist ICI 182,780 (500 nM) (Tocris Bioscience, Bristol, UK). Queens One tab was used as positive control in this study and it constituted 200 mg of red clover extract in tablet (360 mg) and its active compounds are isoflavones. It is commonly used for the improvement of the symptoms about menopause such as hot flush, night sweats, emotional lability, agitation, and insomnia. After incubation for 6 days, cell viability was evaluated using Ez-Cytox solution (Daeil Lab Service, Seoul, South Korea). Briefly, a 1/10 dose Ez-Cytox solution was added to each well. After incubation for 2 h, optical densities were measured at 450 nm using a multi-microplate reader (SPARK 10 M; Tecan Group Ltd., Männedorf, Switzerland). Cell viability was calculated based on the ratio of 100% of the vehicle group.

### 2.5. Experimental Animals and Experimental Design

All animal studies were performed in accordance with the instructions of the Institutional Animal Care and Use Committee (IACUC) of Gachon University (GIACUC-R2017034, approved 23 February 2018). The experiment was conducted on 36 female Sprague-Dawley rats aged 5 weeks old and weighing 130–150 g (Doo Yeol Biotech, Seoul, Korea). The rats were maintained in a room at 24 °C ± 2 °C with a relative humidity of 50–55%. Food and water were provided ad libitum. After 1 week of acclimatization, all rats were anesthetized with an intraperitoneal injection of Zoletil^®^ (Virbac, Carros, France) and Rompun^®^ (TS, Bayer, Leverkusen, Germany) mixture (3:1, 0.2 mg/kg body weight). Sham operation was performed on six rats (sham) and bilateral ovariectomy (OVX) was performed on the remaining 30 rats. After surgery, the rats were fed a solid diet for 1 week of recovery. Ovariectomized rats were divided into three groups (*n* = 10): (1) OVX, (2) OVX + FeQ, and (3) OVX + VFE. Animals from the OVX + FeQ group were orally administered 100 mg/kg Feramin Q (FeQ) suspension in 0.5% methylcellulose (MC). The animals from the OVX + VFE group were orally treated with 50 mg/kg VFE in 0.5% MC. The animals from sham and OVX groups were administered the same volume 0.5% MC solution for 8 weeks. In order to investigate the effect of VFE on the menopausal model, animal weight was evaluated once a week and blood and the uteruses were collected at the end of the experimental period. The dose of VFE has been determined by a previous study and the dose of FeQ is the concentration calculated for animals based on the intake of menopausal women [26].

### 2.6. Blood Biochemistry

At the end of the experimental period, blood samples were collected in test tubes containing 0.18 M ethylenediaminetetraacetic acid (EDTA) via the inferior vena cava after fasting for 24 h. The plasma was separated by centrifugation at 3500 rpm for 15 min at 4 °C and stored at −20 °C until analysis. The levels of aspartate aminotransferase (AST), alanine aminotransferase (ALT), creatinine, and total cholesterol were measured using GENIA (Seong-Nam, Korea). In addition, estradiol levels were measured using commercially available Quantikine^®^ immunoassay kits (R&D Systems, Minneapolis, MN, USA).

### 2.7. Uterus Histological Analysis

The uterus was excised from experimental animals. The tissue weight was measured with cooling saline and photographed. Some uterine tissues were fixed in 10% neutral buffered formalin for 24 h. Specimens were embedded in paraffin and 5 μm sections were prepared and stained with hematoxylin and eosin (H&E). Uterine tissue slides were examined under a light microscope and the endometrium thickness was confirmed. Tissue samples were assessed at 40× and 100× magnifications and photographed using an Olympus microscope (model: BX43F, Tokyo, Japan).

### 2.8. Western Blot Analysis

The uterus proteins were loaded at 30 μg into each lane of a 10% (mg/mL) sodium dodecyl sulfate-polyacrylamide electrophoresis (SDS-PAGE) gel. After gel electrophoresis, the protein bands were transferred to polyvinylidene fluoride (PVDF) membranes (Millipore, Billerica, MA, USA), which were then blocked with 2% (mg/mL) bovine serum albumin (BSA) and probed with appropriately diluted (1:1000) mouse monoclonal ER-α primary antibody (Santa Cruz, Dallas, TX, USA, sc-8005), mouse monoclonal ER-β primary antibody (Santa Cruz, Dallas, TX, USA, sc-53494), and rabbit monoclonal GAPDH primary antibody (Cell Signaling Technology, Danvers, MA, USA) in 2% BSA with 0.1% (mL/mL) Tween-20 in Tris-buffered saline (TBS) overnight at 4 °C. Next, a 1:2000 diluted goat anti-mouse secondary antibody conjugated with peroxidase (Santa Cruz, Dallas, TX, USA) in 2% BSA with 0.1% (mL/mL) Tween-20 in TBS was incubated with the membrane for 1 h at room temperature. In order to visualize the immunoreactive bands, the membranes were reacted with SuperSignal West Femto Maximum Sensitivity Substrate (Thermo Scientific, Rockford, IL, USA). The density of the protein band was quantified using image J software normalized by GAPDH expression.

### 2.9. Sample Preparation for HPLC Analysis

All samples for analysis were dissolved in methanol. All standard compounds were dissolved to a concentration of 1 mg/mL for stock solutions. Diluted solutions were subsequently prepared to generate a calibration curve of standard solutions at various concentrations from 7.8125 to 500 μg/mL for artemetin (1), vitexicarpin (2), and vanillic acid (6). *V. rotundifolia* extract was prepared at a concentration of 10 mg/mL. All analytical solutions were filtered using a 0.45 µm RC-membrane syringe filter (Sartorius, Göttingen, Germany).

### 2.10. Optimization of Chromatographic Conditions

An Agilent 1260 Infinity HPLC system with a G1311C quaternary pump, a G1329B auto sampler, a G1316A column oven, a G1315D photodiode array (PDA) detector, and Agilent ChemStation software (Agilent Technologies, Santa Clara, CA, USA) was used for HPLC analysis. A ZORBAX SB-C18 (250 mm × 21.2 mm I.D., 5 µm, Agilent Technologies) column was used for chromatography at 30 °C with UV detection at 280 nm. The mobile phase comprised water containing 0.2% formic acid (solvent A) and acetonitrile (solvent B) with gradient elution of 0–20 min, 8% B; 20–25 min, 8–25% B; 25–28 min, 25–27% B; 28–35 min, 27–37% B; 35–40 min, 37–40% B; 40–52 min, 40% B; 52–60 min, 40–60% B; 60–65 min, 65–100% B at a flow rate of 0.8 mL/min. Before injection of the next sample, the column was re-equilibrated with the initial gradient of the solvents for 7 min.

The analytical method was optimized by adjusting chromatographic parameters such as solvent, column, gradient range of elution, flow rate, column temperature, mobile phase, and detection wavelength. Acidic water (0.2% formic acid, *v*/*v*) and acidic acetonitrile (0.2% formic acid, *v*/*v*) were used as the stationary phase to elute C18 column. A variety of compounds in the extract were scanned using photodiode array (PDA) detectors in the wavelength range of 190–400 nm. A wavelength of 280 nm was deemed suitable for detection because it produced much better resolution and baseline in the chromatogram than others. The best method was optimized for chromatographic separation. Chromatogram analysis was performed from the ascending, apex, and descending regions as well as for the symmetry of peaks. As we had six compounds isolated from VFE, each peak on the chromatogram was identified by the spiking method and was consistent with MS results. In the HPLC chromatogram, two peaks (at 15.8 and 47.0 min) corresponded to vitexicarpin (**2**) and vanillic acid (**6**), respectively. The compounds in the extract were quantitatively analyzed and calibration curves were obtained by plotting the peak area versus the concentration for each analyte by least-square regression analysis. The calibration equation for each analyte was obtained using seven levels of concentrations ranging from 0.0078 to 0.5 mg/mL. The range of all calibration curves was adequate for the simultaneous analysis of three compounds in the sample extract. The linear correlation coefficients (*r*^2^) of the calibration curves were higher than 0.99, indicating good linearity.

### 2.11. Statistical Analysis

Statistical analysis was conducted using the GraphPad Prism, version 5.0 (GraphPad software Inc, La Jolla, CA, USA). In the experiment, quantitative data are expressed as mean ± standard error mean (SEM) for at least three independent experiments. Normal distribution of the data was analyzed using D’Agostino and Pearson omnibus normality test. Statistical significance was evaluated and determined by one-way analysis of variance (ANOVA), followed by Tukey’s post hoc test. Individual differences between groups were evaluated statistically significant at *p* < 0.05.

## 3. Results

### 3.1. Estrogenic Effects of Various Polar Extracts of V. rotundifolia on MCF-7 Cells

**Figure 1:**
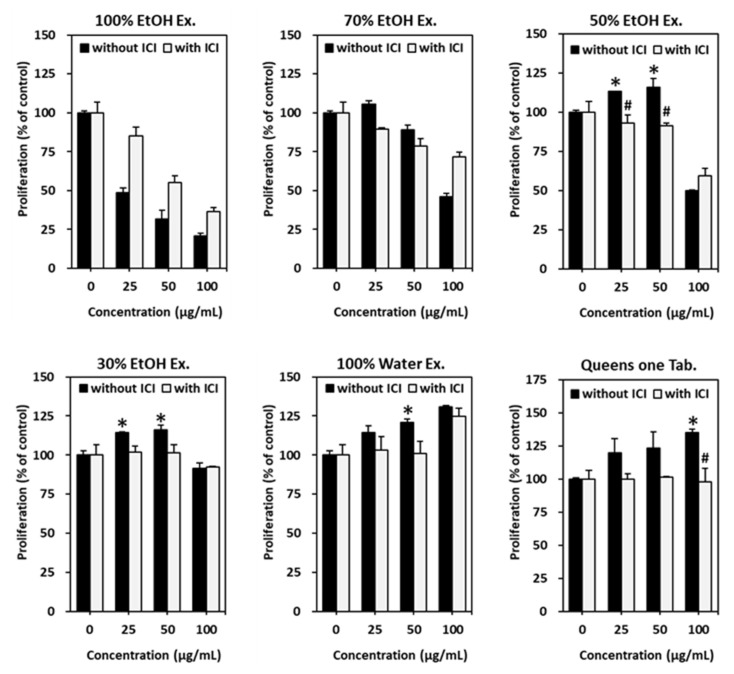
The estrogenic effect of *V. rotundifolia* fruits extracts on MCF-7 cell proliferation. Queens One Table was used as the positive control. The data are represented as the mean ± standard error mean (SEM). Statistical significance was evaluated and determined by a one-way analysis of variance (ANOVA), followed by the Tukey’s post-hoc test. Differences between means were considered statistically significant when *p* < 0.05. * *p* < 0.05 vs. untreated cells. ^#^ *p* < 0.05 vs. each group without ICI 182,780 treatment. 100% EtOH Ex.: Ethanol extract; 70% EtOH Ex.: 70% ethanol extract; 50% EtOH Ex.: 50% ethanol extract; 30% EtOH Ex.: 30% ethanol extract; Water Ex.: distilled water extract; ICI: ICI 182,780.

We assessed the effects of various polar extracts of *V. rotundifolia* fruits on the ER-positive MCF-7 human breast cancer cell line. MCF-7 cells were exposed to different extracts in the absence of other hormones for 6 days. Queens One Table, which is a commercial drug, was used as a positive control. As shown in Figure 1, treatment with Queens One Table increased MCF-7 cell proliferation in a concentration-dependent manner up to 100 μg/mL. The increased proliferation of cells was significantly suppressed by the ER antagonist ICI 182,780. Therefore, the cell proliferation effect of Queens One Table was estrogen-dependent. Among the various polar extracts, 30% and 50% ethanol extracts significantly increased cell proliferation at 50 μg/mL concentration. This effect was suppressed by co-treatment with ICI 182,780. The estrogenic activity of 30% and 50% ethanol extracts was evident at 25 and 50 μg/mL concentrations, respectively. Although 50% ethanol extract was cytotoxic at 100 μg/mL, the significance was displayed for estrogenic activity. Thus, 50% ethanol extract was regarded as a good agonist against ER as compared with 30% ethanol extract.

### 3.2. Analysis of the Compounds in the Extract by HPLC-UV

**Figure 2:**
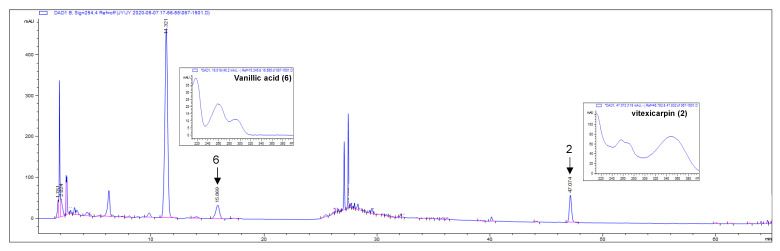
HPLC-UV/PDA chromatogram of 30% aqueous EtOH extract of *V. rotundifolia* fruit.

The contents of compounds **2** and **6**, measured based on calibration curves, were 1.981 ± 0.019 and 1.049 ± 0.004 mg/g, respectively (Table 1). Other compounds were not analyzed because their amounts were very low (Figure 2).

### 3.3. Estrogenic Effects of the Constituents of the Extract on MCF-7 Cells

**Figure 3:**
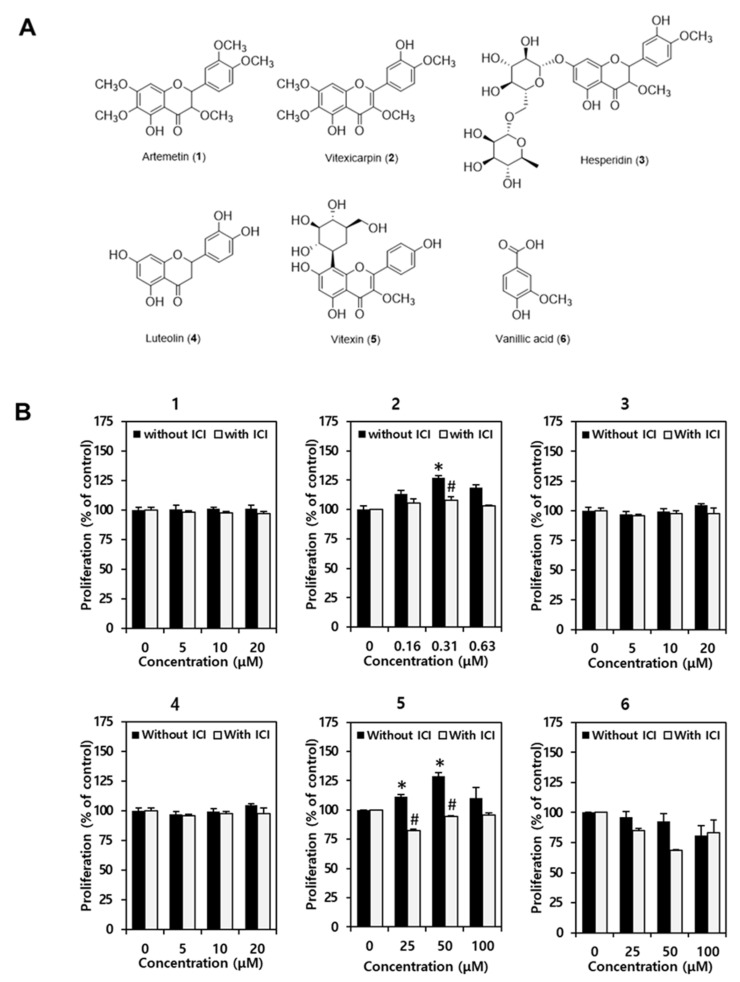
Structures of compounds **1**–**6** isolated from the fruits of *V. rotundifolia*. Artemetin (**1**), vitexicarpin (**2**), hesperidin (**3**), luteolin (**4**), vitexin (**5**), and vanillic acid (**6**) (**A**). The estrogenic effects of the constituents from *V. rotundifolia* fruits on MCF-7 cell proliferation (**B**). The data are represented as the mean ± standard error mean (SEM). Statistical significance was evaluated and determined by a one-way analysis of variance (ANOVA), followed by the Tukey’s post-hoc test. Differences between means were considered statistically significant when *p* < 0.05. * *p* < 0.05 vs. untreated cells. ^#^ *p* < 0.05 vs. each group without ICI 182,780 treatment. ICI: ICI 182,780.

In order to compare the estrogenic activities of the constituents of *V. rotundifolia* fruits (Figure 3A), we investigated the proliferation of MCF-7 cells in the presence of each constituent. The cells were exposed to each constituent in the absence of and antagonist ICI 182,780 for 6 days. As shown in Figure 3, treatment with vitexin (**5**) at 25 and 50 μM concentrations increased MCF-7 cell proliferation to 111.5 ± 1.9 and 129.4 ± 3.4, respectively. Vitexicarpin (**2**) increased MCF-7 cell proliferation to 113.3 ± 3.1 and 121.2 ± 1.8 at low concentrations of 0.16 and 3.1 μM, respectively. This effect was completely attenuated by co-treatment with ICI 182,780. As vitexin (**5**) and vitexicarpin (**2**) exerted significant estrogenic activities, we consider them as agonists of ER. The other constituents had no cell proliferative effect. Vitexin and vitexicarpin are, therefore, suggested as the active constituents of *V. rotundifolia* fruits.

### 3.4. Effects of VFE on the Body Weight and Cholesterol of Ovariectomized Rats

**Figure 4:**
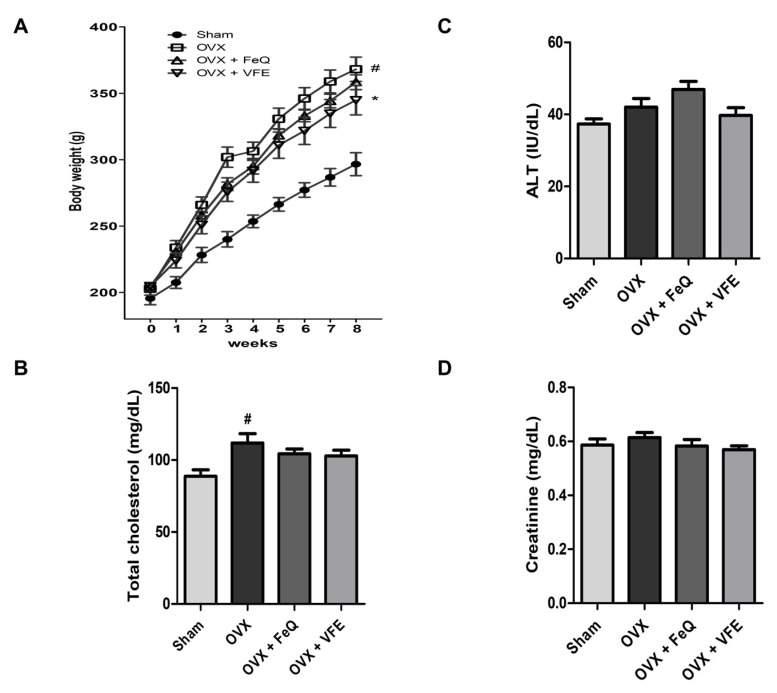
Effects of VFE on the body weight, cholesterol level, and liver and kidney toxicity markers of ovariectomized (OVX) rats. (**A**) Change in body weight. (**B**) Levels of plasma cholesterol. (**C**) Plasma ALT levels. (**D**) Plasma creatinine levels. The data are expressed as mean ± SEM. Statistical significance was evaluated and determined by a one-way analysis of variance (ANOVA), followed by the Tukey’s post-hoc test. ^#^ *p* < 0.05 vs. sham, * *p* < 0.05 vs. OVX.

We investigated the effect of VFE on body weights of test animals after administration. The body weights of the rats from the OVX group were significantly higher than those of the rats from the sham group. The body weight gain in the VFE group was significantly lower than that in the OVX group (Figure 4A). In addition, the level of cholesterol in the OVX group was significantly higher than that in the sham group but tended to decrease in FeQ-treated and VFE-treated groups (Figure 4B).

### 3.5. Effects of VFE on the Plasma Biomarkers of Toxicity in Ovariectomized Rats

In order to confirm the possible toxicity of VFE to the liver and kidney, blood samples were collected after the experimental period and levels of toxicity markers were analyzed. Among hepatotoxicity markers, plasma ALT levels tended to increase in OVX-treated and FeQ-treated rats but not in the rats from the VFE group (Figure 4C). The levels of plasma creatinine, which is a nephrotoxicity marker, also tended to increase but not significantly in the OVX group as compared to those in the sham group, but not in FeQ and VFE treatment groups (Figure 4D).

### 3.6. Estrogenic Effect of VFE on the Uterus of Ovariectomized Rats

**Figure 5:**
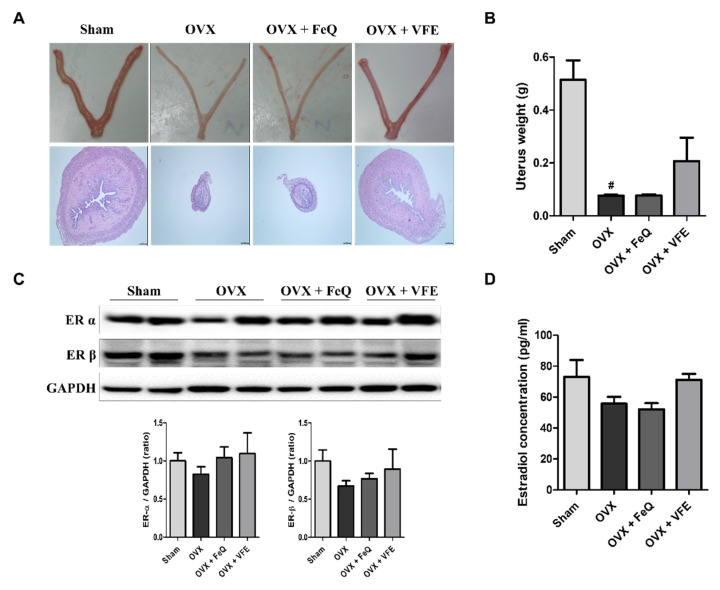
Estrogenic effect of VFE on the uterus of ovariectomized rats. (**A**) The uterus tissue and micrographs of H&E-stained samples (200×), (**B**) uterus tissue weight, (**C**) protein expression levels of estrogen receptor-α and receptor-β, and (**D**) plasma estradiol level. The data are expressed as means ± SEM. Statistical significance was evaluated and determined by a one-way analysis of variance (ANOVA), followed by the Tukey’s posthoc test. ^#^ *p* < 0.05 vs. sham.

In order to evaluate the estrogenic effect of VFE, the uterine tissue from each group was collected after the end of the experimental period and analyzed for atrophy and ER expression. Images of the uterus tissue and histological uterine sections revealed atrophy in the uterus and reduction in the uterine weight in animals from the OVX group as compared with those in the animals from the sham group. VFE treatment ameliorated uterine atrophy (Figure 5A), but the weight of the uterus was not statistically different from OVX group (Figure 5B) and this is evident from the thickening of the endometrium. In addition, we evaluated ER expression in the uterine tissue by western blotting. The protein expression levels of ER-α and ER-β were lower in the OVX group than in the sham group. However, the expressions of ER-α and ER-β in the VFE-administered group were not statistically different from that in the OVX group (Figure 5C). Furthermore, enzyme-linked immunosorbent assay (ELISA) analysis of estradiol using plasma samples revealed the decrease in estradiol levels in the OVX group as compared with that in the sham group. However, estradiol levels were restored after VFE administration (Figure 5D).

## 4. Discussion

Flavonoids act similar to female hormones and are beneficial to menopausal women [17,18,19,20,21]. *V. rotundifolia* is known to contain many flavonoids, which may be beneficial to menopausal women. We confirmed the estrogen-like activity of the VFE and its compounds in MCF-7 cells using E-screen assay and, further, the beneficial estrogenic effect of VFE in the animal experiment using the ovariectomized rat.

Estrogen is known to stimulate the proliferation of MCF-7 cells. The E-Screen assay determines the estrogen-like activity of different compounds [4,27] and is one of the most reliable methods for confirming the estrogen-like activity of molecules, including cell growth and estrogen-related expression of genes related to the estrogen signaling pathway [28,29]. Moreover, the proliferative effects of phytoestrogens on MCF-7 cells have been reported in other studies, including reports on genistein [30,31]. In the present study, the estrogen-like activity of VFE was confirmed using MCF-7 cells. In our study, 30% and 50% ethanol extracts exhibited estrogenic activities at concentrations of 25 and 50 μg/mL. However, 50% ethanol extract was cytotoxic at a concentration of 100 μg/mL. In the experiment conducted to confirm the active constituents of *V. rotundifolia* fruits, treatment with 25 and 50 μM vitexin and 25 μM luteolin resulted in an increase in the proliferation of MCF-7 cells. Vitexin, but not luteolin, could completely attenuate the effect of ICI 182,780, which is an ER antagonist. Thus, we suggest that 30% ethanol extract is a good agonist for ER and that vitexin is the active constituent that acts on ER. We thought that small amounts of other compounds with estrogenic effects, as well as vitexin, may be present in *V. rotundifolia* fruits extracts. The presence of other compounds supports our thinking in other studies evaluating the estrogen-like activity against *V. rotundifolia* fruits extracts [32,33].

Ovariectomy is a surgical procedure that induces menopause in experimental animals. Ovariectomized female rats show dramatic menopausal syndrome after cessation of ovarian function [34,35]. Our study found that ovariectomized rats had significantly increased body weight, as reported in other studies. In menopausal women, hormonal changes increases abdominal obesity [36]. In the present study, we show that VFE treatment alleviated the body weight gain and blood cholesterol levels reported in untreated OVX rats. In addition, the levels of hepatotoxicity markers AST and ALT and nephrotoxicity markers creatinine were similar or slightly lower in our study. We believe that VFE administration is not toxic to the liver and kidneys. Thus, the effects on body weight and blood cholesterol following VFE treatment were associated with its estrogen-like activity. In addition, we observed uterine atrophy, which is a symptom of estrogen insufficiency. Previous studies have reported that uterine weight loss is related to the reduction in uterine epithelial height, uterine myometrial thickness, and uterine stromal expansion [37]. In our experiment, uterine atrophy was observed in ovariectomized animals. However, VFE treatment ameliorated uterine atrophy and tissue weight loss observed in untreated OVX rats. A previous study has shown that phytoestrogens such as genistein and daidzein can alter endometrial stromal cells and interfere with decidualization signaling [38]. We also confirmed that VFE increased the protein expression of ER-α and blood estradiol levels. These results indicate that the biological activity of VFE is associated with its function as an estrogen agonist, wherein it mimics the action of endogenous estrogen or with the altered synthesis and metabolic patterns of endogenous hormones [39,40].

Regarding the structure–activity relationship (SAR), flavone-type compounds with double bond between C-2 and C-3 of the flavonoid skeleton showed more potent activity than flavanone-type compounds with saturated C-2 and C-3. In addition, methoxylation at C-3 and hydroxylation of B ring in the flavone skeleton seem to be important for the activity as shown in compounds **2** and **5**. Glycosylation in the A ring of flavonoid decreased the activity (comparing **2** with **5**). In case of luteolin with catechol moiety in B ring of the flavonoid, it was excluded due to its toxicity although it exhibited potent proliferative activity.

We have previously analyzed the contents of different compounds in VFE [41]. In our previous study, the fruits were extracted with 100% methanol (MeOH). However, 30% aqueous EtOH was used for extraction in the present study owing to the application of this extract in animals. The differences in the extraction solvents used may have contributed to the variations in the detected compounds and their contents. Vitexicarpin (**2**) was better extracted with 100% MeOH than with 30% aqueous EtOH. The active compound vitexin (**3**) was not detected in both 100% MeOH and 30% aqueous EtOH extracts owing to its presence in a low amount. However, the content of vitexin in the extract is presumed to be 0.2 mg/g based on the amounts isolated from the extracts in our study.

## 5. Conclusions

In summary, our experiment identified the active constituents in the polar extract of VFE with estrogen-like activity on MCF-7 cells. Vitexin and vitexicarpin are suggested as the active constituents of *V. rotundifolia* fruits. In animal experiments, the estrogen-like effect of VFE was confirmed from the amelioration of the uterine atrophy, increase in blood estradiol and ER expression, and the alleviation of body weight gain. Therefore, the potential of *V. rotundifolia* to improve the symptoms of menopausal women was confirmed and the mechanism of action of active ingredients on ER was revealed.

## Figures and Tables

**Table 1:** Calibration curve and contents of compounds in *V. rotundifolia* fruit.

Analytes	Calibration Curve	*r* ^2^	Content (mg/g)
Vitexicarpin (2)	y = 4,670,666.3799x + 28.1243	0.9999	1.981 ± 0.019
Vanillic acid (6)	y = 6,577,450.3226x + 99.4849	0.9994	1.049 ± 0.004
